# Association of impaired sensitivity to thyroid hormones with hyperuricemia through obesity in the euthyroid population

**DOI:** 10.1186/s12967-023-04276-3

**Published:** 2023-07-05

**Authors:** Zhiyuan Wu, Yue Jiang, Pingan Li, Yutao Wang, Haiping Zhang, Zhiwei Li, Xia Li, Lixin Tao, Bo Gao, Xiuhua Guo

**Affiliations:** 1 Beijing Municipal Key Laboratory of Clinical Epidemiology, School of Public Health, Capital Medical University, No.10 Xitoutiao, Youanmen Street, Beijing, 100069 China; 2 Centre for Precision Health, School of Medical and Health Sciences, Edith Cowan University, 270 Joondalup Drive, Joondalup, WA 6027 Australia; 3 Shanghai Fufan Information Technology Co., Ltd, No.323 Guoding Road, Yangpu District, Shanghai, 200433 China; 4 Department of Mathematics and Statistics, La Trobe University, Melbourne, 3086 Australia; 5 Department of Epidemiology and Health Statistics, School of Public Health, Capital Medical University, No.10 Xitoutiao, Youanmen Street, Beijing, 100069 China

**Keywords:** Thyroid hormones sensitivity, Hyperuricemia, Uric acid, Body mass index (BMI), Mediation analysis

## Abstract

**Background:**

Impaired sensitivity to thyroid hormones is a newly proposed clinical entity associated with hyperuricemia in the subclinical hypothyroid population. However, it is unknown whether the association exists in the euthyroid population. This study aimed to explore the association of impaired sensitivity to thyroid hormones (assessed by the thyroid feedback quantile-based index [TFQI], parametric thyroid feedback quantile-based index [PTFQI], thyrotrophic thyroxine resistance index [TT4RI] and thyroid-stimulating hormone index [TSHI]) with hyperuricemia and quantify the mediating effect of body mass index BMI in the euthyroid population.

**Methods:**

This cross-sectional study enrolled Chinese adults aged ≥ 20 years who participated in the Beijing Health Management Cohort (2008–2019). Adjusted logistic regression models were used to explore the association between indices of sensitivity to thyroid hormones and hyperuricemia. Odds ratios [OR] and absolute risk differences [ARD] were calculated. Mediation analyses were performed to estimate direct and indirect effects through BMI.

**Results:**

Of 30,857 participants, 19,031 (61.7%) were male; the mean (SD) age was 47.3 (13.3) years; and 6,515 (21.1%) had hyperuricemia. After adjusting for confounders, individuals in the highest group of thyroid hormone sensitivity indices were associated with an increased prevalence of hyperuricemia compared with the lowest group (TFQI: OR = 1.18, 95% CI 1.04–1.35; PTFQI: OR = 1.20, 95% CI 1.05–1.36; TT4RI: OR = 1.17, 95% CI 1.08–1.27; TSHI: OR = 1.12, 95% CI 1.04–1.21). BMI significantly mediated 32.35%, 32.29%, 39.63%, and 37.68% of the associations of TFQI, PTFQI, TT4RI and TSHI with hyperuricemia, respectively.

**Conclusions:**

Our research revealed that BMI mediated the association between impaired sensitivity to thyroid hormones and hyperuricemia in the euthyroid population. These findings could provide useful evidence for understanding the interaction between impaired sensitivity to thyroid hormone and hyperuricemia in euthyroid individuals and suggest the clinical implications of weight control in terms of impaired thyroid hormones sensitivity.

**Supplementary Information:**

The online version contains supplementary material available at 10.1186/s12967-023-04276-3.

## Keypoints

**Quetions** To what extent is impaired sensitivity to thyroid hormones associated with hyperuricemia through body mass index (BMI) in the euthyroid population?

**Findings** In this cross-sectional study of 30,857 euthyroid adults, impaired sensitivity to thyroid hormones was independently associated with an increased prevalence of hyperuricemia. BMI mediated 32.3–39.6% of the association between impaired sensitivity to thyroid hormones and hyperuricemia.

**Meaning** This study revealed that BMI mediated the association between impaired sensitivity to thyroid hormones and hyperuricemia in the euthyroid population, suggesting the potential clinical benefit of weight control in maintaining metabolic health among individuals with impaired thyroid hormones sensitivity.

**Supplementary Information:**

The online version contains supplementary material available at 10.1186/s12967-023-04276-3.

## Introduction

Hyperuricemia, a metabolic state characterized by elevated serum uric acid levels, can lead to gout [1]. Due to changes in dietary factors and lifestyles, the prevalence of gout is increasing globally, posing a serious public health concern [2]. Epidemiological data indicate that hyperuricemia is more prevalent in high-income countries than in developing regions [3–5]. The National Health and Nutrition Examination Survey reported that the prevalence of hyperuricemia among US adults was 20.1% during 2015–2016 [6]. A Chinese health survey found that the prevalence of hyperuricemia among individuals aged 20–80 was 25.1% in males and 15.9% in females [7]. In addition to gout, hyperuricemia is an independent risk factor for metabolic diseases (such as diabetes, metabolic syndrome, and hyperlipidemia), chronic kidney disease and cardiovascular diseases [8–12]. Therefore, it is crucial to identify the related risk factors and high-risk individuals for hyperuricemia.

Thyroid hormones play an important role in regulating biological metabolism, and free thyroxine (FT4) and thyroid-stimulating hormone (TSH) are regulated by a negative feedback mechanism in the hypothalamic-pituitary-thyroid axis. We searched PubMed and Web of Science with the terms “(thyroid hormones sensitivity OR thyroid hormones) AND (metabolic health OR metabolic disorder)” for papers published from database inception to Dec 31, 2022. The separate relationships of TSH and FT4 with hyperuricemia have been reported. We found that previous studies have reported the associations between hypothyroidism and various metabolic disorders, including dyslipidemia [13, 14], obesity [15], diabetes [16, 17] and hyperuricemia [18, 19]. However, the reported relationships between FT4 or TSH alone and hyperuricemia were inconsistent. Thyroid hormones sensitivity is a newly proposed functional entity that takes into account both FT4 and TSH levels. Both high FT4 and high TSH are present in the resistance to thyroid hormones syndrome, reflecting energy balance problems. It was first reported by Refetoff et al. [20] and is characterized by elevated serum levels of FT4 and free triiodothyronine accompanied by normal or slightly elevated thyrotropin [21]. Subsequently, researchers have proposed indices representing impaired sensitivity to central thyroid hormones, including the thyroid feedback quantile-based index [TFQI] [22], parametric thyroid feedback quantile-based index (PTFQI) [22], thyrotrophic thyroxine resistance index (TT4RI) [23] and thyroid-stimulating hormone index (TSHI) [24]. Previous studies have shown that impaired thyroid hormones sensitivity is associated with prediabetes [25], diabetes [22, 26], cardiovascular disease [27], metabolic syndrome [22], obesity [27], hypertension [28], nonalcoholic fatty liver [29], hyperhomocysteinaemia [30] and osteoarthritis [31]. Notably, Sun et al. [27] reported that impaired sensitivity to thyroid hormones is associated with a higher risk of hyperuricemia in patients with subclinical hypothyroidism. However, further studies are needed to determine whether impaired sensitivity to thyroid hormones is associated with hyperuricemia in the euthyroid population. Currently, there is a lack of interventions to address impaired sensitivity to thyroid hormones. It is noteworthy that mediation analysis can reveal indirect pathways for potential intervention strategies, particularly through nonpharmaceutical and modifiable factors such as lifestyle changes [32, 33].

In this study, we aimed to investigate the relationship between impaired sensitivity to thyroid hormones and hyperuricemia in the euthyroid population and quantify the direct and indirect associations by body mass index (BMI), thus providing potential interventions for those with impaired thyroid hormones sensitivity to achieve metabolic health status from the perspective of energy balance.

## Methods

### Study population and design

This study is a secondary analysis using data from the Beijing Health Management Cohort (BHMC), which is a dynamic cohort designed to investigate the risk factors and biomarkers of cardiovascular and metabolic diseases [34]. Participants who underwent a health examination between 2008 and 2019 were primarily included in this current study. Individuals with missing data on uric acid (n = 355), thyroid dysfunction (n = 7,635), using thyroid hormones medication (n = 63) or younger than 20 years (n = 21) were excluded. Finally, a total of 30,857 participants were included in this cross-sectional study (Additional file 1: Figure S1). The study was conducted in accordance with the principles of the Declaration of Helsinki and was approved by the Ethics Committee of Capital Medical University (approval number: 2020SY031). This study followed the Strengthening the Reporting of Observational Studies in Epidemiology (STROBE) reporting guidelines [35]. Data were analysed from May to November 2022.

### Data collection and definition

Demographic characteristics (age, sex and education), lifestyles (physical activity, current smoking and current drinking) and health-related information (disease diagnosis history and medication use) were collected by our trained staff using a standard questionnaire. Education level was classified as primary (illiterate or primary school), secondary (middle school or high school), or tertiary (bachelor’s degree or above). Active physical activity was defined as “engaging in moderate to vigorous physical activity at work or during leisure time more than 4 times and 80 min per week”. Self-reported disease history included hypertension, diabetes and thyroid dysfunction. Physical examination included measurements of height, weight and blood pressure. Body mass index (BMI) was calculated as weight in kilograms divided by height in meters squared, which was available among 28,153 participants. Obesity was defined as a BMI ≥ 28.0 kg/m^2^ for the Chinese population [36]. Systolic blood pressure and diastolic blood pressure were measured on the right arm using a sphygmomanometer after at least 10 min of rest and were calculated as the average of two measurements. Concentrations of fasting glucose and serum uric acid were tested before breakfast after overnight fasting (no food, except drinking water for at least 8 h) using an automatic biochemical analyser (Roche Cobas c 701). Serum TSH and FT4 levels were measured using electrochemiluminescence immunoassay (ECLIA) on an autoanalyzer (Mindray CL-2000i). The assay-specific reference ranges for TSH and FT4 were 0.35–5.00 mIU/L and 11.20–23.81 pmol/L (to convert FT4 to ng/dL, divided by 12.871), respectively.

Hypertension was defined as systolic blood pressure ≥ 140 mmHg or diastolic blood pressure ≥ 90 mmHg or self-reported diagnosis history of hypertension or use of any anti-hypertensive medication [37]. Diabetes was defined as fasting glucose ≥ 7.0 mmol/L or self-reported diagnosis history of diabetes or use of any hypoglycemic medication [38]. Euthyroid was defined as serum TSH and FT4 levels within the normal ranges and no use of thyroid hormone medication [39]. Hyperuricemia was defined as serum uric acid ≥ 360 μmol/L in females and ≥ 420 μmol/L in males or the use of uric acid-lowering medication [40].

### Definition of thyroid hormone sensitivity

The thyroid feedback quantile-based index (TFQI) was calculated as the empirical cumulative distribution function (cdf) FT4-(1-cdf TSH) [22]. We also calculated the parametric thyroid feedback quantile-based index (PTFQI) [22] using the standard normal cumulative distribution function: Φ((FT4-μFT4)/σFT4)-(1-Φ((Ln TSH-μLn TSH)/σLn TSH)). In our population, μFT4 was 15.869, σFT4 was 2.323, μLn TSH was 0.640 and σLn TSH was 0.482. The TFQI and PTFQI values range from -1 to 1, with higher positive values indicating more impaired sensitivity to thyroid hormones. The thyrotrophic thyroxine resistance index (TT4RI) was calculated as FT4 (pmol/L) × TSH (mIU/L) [23]. The thyroid-stimulating hormone index (TSHI) was calculated as Ln TSH (mIU/L) + 0.1345 × FT4 (pmol/L) [24]. Higher values of TT4RI and TSHI indicate a higher degree of impaired sensitivity to thyroid hormones.

### Statistical analysis

Normally distributed variables are expressed as the mean (standard deviation), skewed variables are expressed as the median [interquartile range], and categorical variables are expressed as frequencies (proportions). Continuous variables were compared using Student’s t test or the Mann–Whitney U test. Categorical variables were compared using the chi-square test. The distributions of thyroid hormone sensitivity indices were presented using violin plots.

Logistic regression models were used to investigate the relationship between impaired sensitivity to thyroid hormones and hyperuricemia. The odds ratio (OR) and absolute risk difference (ARD) along with the 95% confidence interval (CI) were calculated [41]. Age, sex, education level, physical activity, current smoking, current drinking, hypertension and diabetes were adjusted. The unadjusted and adjusted dose–response relationships between thyroid hormone sensitivity indices and hyperuricemia were presented using a restricted cubic spline function using 3 knots at the 10^th^, 50^th^ and 90^th^ percentiles. Sensitivity analyses were performed among 28,153 participants with available BMI data.

We conducted a mediation analysis to assess the direct and indirect associations between impaired sensitivity to thyroid hormones and hyperuricemia via BMI among 28,153 participants. Thyroid hormone sensitivity indices were analysed both as continuous variables after normalization and as categorical variables. In brief, thyroid hormone sensitivity indices were used as predictor variables (X), BMI as a mediator (M) and hyperuricemia as the outcome variable (Y). The analysis included four steps: (1) establishing that X is associated with Y (Model Y = β_Tot_ X) (β_Tot_ = total effect); (2) establishing that X is associated with M (Model M = β_1_ X) (β_1_ = indirect effect1); (3) determining which part of Y is explained by controlling for X (Model Y = β_2_ M + β_Dir_ X) (β_2_ = indirect effect, β_Dir_ = direct effect); and (4) calculating the proportion of indirect or mediation effect: mediation effect (%) = (β_1_ × β_2_/β_Tol_) × 100%. This method has been widely used in previous studies to quantify the mediating effect [42, 43].

All statistical analyses were performed using R software (version 4.2.1). Mediation analysis was performed using the ‘mediation’ package. A two-sided P value < 0.05 was considered statistically significant.

## Results

### Characteristics

Of 30,857 participants, 19,031 (61.7%) were males; the mean (SD) age was 47.3 (13.3) years. A total of 6515 participants had hyperuricemia (21.1%). Individuals with hyperuricemia were more likely to be male, current smokers and drinkers, have a higher BMI and FT4 levels, and have hypertension (Table 1). Thyroid hormones sensitivity indices (TFQI, PTFQI, TT4RI and TSHI) were significantly higher in the hyperuricemia group than in the nonhyperuricemia group (all P values < 0.05; Additional file 1: Figure S2). As shown in Additional file 1: Table S1, TSH, FT4 and uric acid concentrations were significantly higher in the obesity group than in those without obesity. Similarly, thyroid hormone sensitivity indices (TFQI, PTFQI, TT4RI and TSHI) were significantly higher in the obesity group (all P values < 0.05; Additional file 1: Figure S2).

**Table 1:** Basic characteristics of the participants

	Overall	Normouricemia	Hyperuricemia^e^	*P* value
Participants, No	30,857	24,342	6515	
Age, mean (SD), y	47.3 (13.3)	47.2 (13.1)	47.7 (14.1)	0.003
Sex				
Female	11,826 (38.3)	10,692 (43.9)	1134 (17.4)	< 0.001
Male	19,021 (61.7)	13,650 (56.1)	5381 (82.6)	
Educational level				
Primary	2823 (9.1)	2230 (9.2)	593 (9.1)	0.085
Secondary	21,920 (71.0)	17,352 (71.3)	4568 (70.1)	
Tertiary	6114 (19.8)	4760 (19.6)	1354 (20.8)	
Active physical activity^a^	12,352 (40.0)	9719 (39.9)	2633 (40.4)	0.484
Current smoking	7949 (25.8)	5979 (24.6)	1970 (30.2)	< 0.001
Current drinking	15,930 (51.6)	12,211 (50.2)	3719 (57.1)	< 0.001
BMI, mean (SD), kg/m^2 b^	25.3 (3.5)	24.8 (3.4)	27.1 (3.5)	< 0.001
Hypertension^c^	6564 (21.3)	4621 (19.0)	1943 (29.8)	< 0.001
Diabetes^d^	3167 (10.3)	2493 (10.2)	674 (10.3)	0.824
TSH, median [IQR], mIU/L	1.9 [1.4, 2.7]	1.9 [1.4, 2.7]	2.0 [1.4, 2.7]	0.613
FT4, median [IQR], pmol/L	15.8 [14.3, 17.4]	15.8 [14.2, 17.4]	16.0 [14.4, 17.6]	< 0.001
UA, mean (SD), μmol/L	340.3 (89.6)	306.9 (63.0)	464.9 (59.0)	< 0.001

*SD* standard deviation, *IQR* interquartile range, *BMI* body mass index, *TSH* thyrotropin, *FT4* free thyroxine, *UA* uric acid

SI conversion factors: To convert FT4 to ng/dL divided by 12.871

^a^Active physical activity refers to having moderate or intense exercise ≥ 80 min a week

^b^BMI is calculated as weight in kilograms divided by height in meters squared. BMI data were available for 28,153 participants

^c^Hypertension was defined as systolic blood pressure ≥ 140 mmHg or diastolic blood pressure ≥ 90 mmHg or self-reported diagnosis history of hypertension or use of any anti-hypertensive medication

^d^Diabetes was defined as fasting glucose ≥ 7.0 mmol/L or self-reported diagnosis history of diabetes or using any glucose-lowering medication

^e^Hyperuricemia was defined as serum uric acid ≥ 360 μmol/L in females and ≥ 420 μmol/L in males or the use of uric acid-lowering medications

### Impaired sensitivity to thyroid hormones and hyperuricemia

**Fig. 1:**
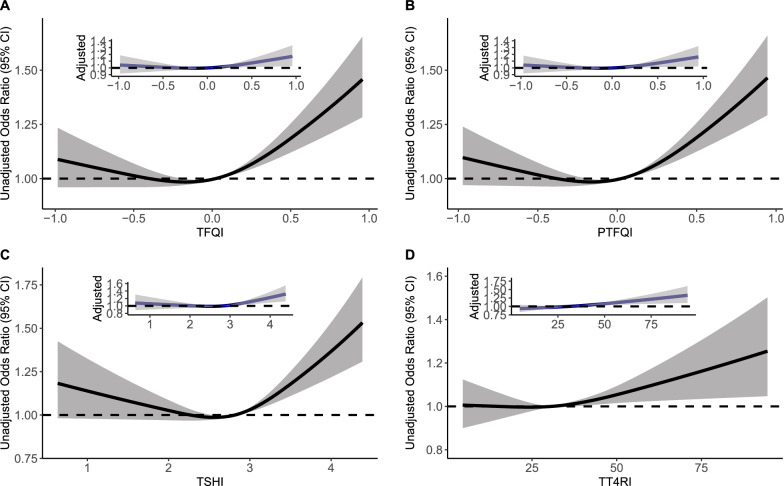
Unadjusted and adjusted dose–response relationship between thyroid hormones sensitivity indices and hyperuricemia using the restricted cubic spline method. A restricted cubic spline regression model was conducted using 3 knots at the 10th, 50th, and 90th percentiles; the results were adjusted for sex, age (continuous), education level (categorical), current smoking (categorical), current drinking (categorical), active physical activity (categorical), hypertension (categorical) and diabetes (categorical). TFQI, thyroid feedback quantile-based index; PTFQI, parametric thyroid feedback quantile-based index; TT4RI, thyrotrophic thyroxine resistance index; TSHI, thyroid-stimulating hormone index

Figure 1 shows the unadjusted and adjusted dose-response relationships between thyroid hormone sensitivity indices and hyperuricemia. Compared to individuals in the lowest group of thyroid hormone sensitivity indices, those in the highest group had a significantly increased prevalence of hyperuricemia (TFQI: OR = 1.18, 95% CI 1.04–1.35; PTFQI: OR = 1.20, 95% CI 1.05–1.36; TT4RI: OR = 1.17, 95% CI 1.08–1.27; TSHI: OR = 1.12, 95% CI 1.04–1.21) (Table 2). The adjusted attributable risk differences (ARDs, 95% CI) were 2.72% (0.53–4.91%), 2.89% (0.73–5.05%), 2.47% (1.22%–3.73%) and 1.79% (0.54%–3.03%), respectively (Table 3). Additional file 1: Figure S3 and Table S2 present the dose–response relationships and ORs of thyroid hormone sensitivity indices with prevalent hyperuricemia among 28,153 participants with available BMI data.

**Table 2:** Odds ratios of sensitivity of thyroid hormone indices to risk of hyperuricemia by logistic regression analysis

	Model 1		Model 2	
	OR (95% CI)	P value	OR (95% CI)	*P* value
TFQI				
Group 1 [−1, 0]	1 [Ref]		1 [Ref]	
Group 2 (0.000, 0.333]	1.048 (0.983–1.116)	0.152	1.019 (0.955–1.089)	0.566
Group 3 (0.333, 0.667]	1.132 (1.049–1.222)	0.001	1.033 (0.955–1.118)	0.419
Group 4 (0.667, 1.000]	1.403 (1.236, 1.594)	< 0.001	1.183 (1.038–1.348)	0.012
PTFQI				
Group 1 [-1, 0]	1 [Ref]		1 [Ref]	
Group 2 (0.000, 0.333]	1.048 (0.983, 1.118)	0.148	1.023 (0.957–1.092)	0.507
Group 3 (0.333, 0.667]	1.125 (1.043, 1.214)	0.002	1.025 (0.948–1.109)	0.528
Group 4 (0.667, 1.000]	1.432 (1.264, 1.622)	< 0.001	1.195 (1.051–1.359)	0.007
TT4RI				
Quartile 1 (~ , 21.733]	1 [Ref]		1 [Ref]	
Quartile 2 (21.733, 30.867]	0.973 (0.900–1.052)	0.498	1.003 (0.926–1.087)	0.935
Quartile 3 (30.867, 42.693]	1.024 (0.948–1.107)	0.545	1.075 (0.992–1.164)	0.077
Quartile 4 (42.693, ~]	1.084 (1.004–1.171)	0.039	1.169 (1.080–1.266)	< 0.001
TSHI				
Quartile 1 (~ , 2.425]	1 [Ref]		1 [Ref]	
Quartile 2 (2.425, 2.814]	0.993 (0.918–1.074)	0.864	1.021 (0.942–1.107)	0.611
Quartile 3 (2.814, 3.173]	1.049 (0.970–1.134)	0.230	1.071 (0.989–1.161)	0.092
Quartile 4 (3.173, ~]	1.145 (1.060–1.237)	< 0.001	1.120 (1.035–1.213)	0.005

Model 1: crude model; model 2: adjusted for sex, age (continuous), education level (categorical), current smoking (categorical), current drinking (categorical), active physical activity (categorical), hypertension (categorical) and diabetes (categorical)

*OR* odds ratio, *CI* confidence interval, *TFQI* thyroid feedback quantile-based index, *PTFQI* parametric thyroid feedback quantile-based index, *TT4RI* thyrotrophic thyroxine resistance index, *TSHI* thyroid-stimulating hormone index

**Table 3:** Absolute risk difference for logistic regression analysis of sensitivity of thyroid hormone indicators and risk of hyperuricemia

	Model 1		Model 2	
	ARD (95% CI)^a^	P value	ARD (95% CI)^a^	*P* value
TFQI				
Group 1	1 [Ref]		1 [Ref]	
Group 2	0.76% (−0.28% to 1.81%)	0.153	0.30% (−0.73% to 1.33%)	0.566
Group 3	2.08% (0.78% to 3.38%)	0.002	0.51% (−0.73% to 1.75%)	0.421
Group 4	6.03% (3.60% to 8.45%)	< 0.001	2.72% (0.53% to 4.91%)	0.015
PTFQI				
Group 1	1 [Ref]		1 [Ref]	
Group 2	0.54% (−0.28% to 1.82%)	0.150	0.35% (−0.68% to 1.38%)	0.508
Group 3	1.97% (0.69% to 3.26%)	0.003	0.39% (−0.83% to 1.62%)	0.529
Group 4	6.41% (4.01% to 8.81%)	< 0.001	2.89% (0.73% to 5.05%)	0.009
TT4RI				
Quartile 1	1 [Ref]		1 [Ref]	
Quartile 2	-0.44% (−1.72% to 0.83%)	0.498	0.05% (−1.17% to 1.27%)	0.935
Quartile 3	0.40% (−0.89% to 1.68%)	0.545	1.12% (−0.12% to 2.36%)	0.077
Quartile 4	1.37% (0.07% to 2.66%)	0.039	2.47% (1.22% to 3.73%)	< 0.001
TSHI				
Quartile 1	1 [Ref]		1 [Ref]	
Quartile 2	-0.11% (−1.38% to 1.16%)	0.864	0.63% (−0.92% to 1.56%)	0.611
Quartile 3	0.78% (−0.50% to 2.06%)	0.230	1.07% (−0.18% to 2.32%)	0.092
Quartile 4	2.29% (0.99% to 3.58%)	< 0.001	1.79% (0.54% to 3.03%)	0.005

Model 1: crude model; model 2: adjusted for sex, age (continuous), education level (categorical), current smoking (categorical), current drinking (categorical), active physical activity (categorical), hypertension (categorical) and diabetes (categorical)

*ARD* absolute risk difference, *CI* confidence interval, *TFQI* thyroid feedback quantile-based index, *PTFQI* parametric thyroid feedback quantile-based index, *TT4RI* thyrotrophic thyroxine resistance index, *TSHI* thyroid-stimulating hormone index

^a^Refers to the pairwise comparison with group 1 or quartile 1 as the reference group

### Mediation analysis through BMI

**Fig. 2:**
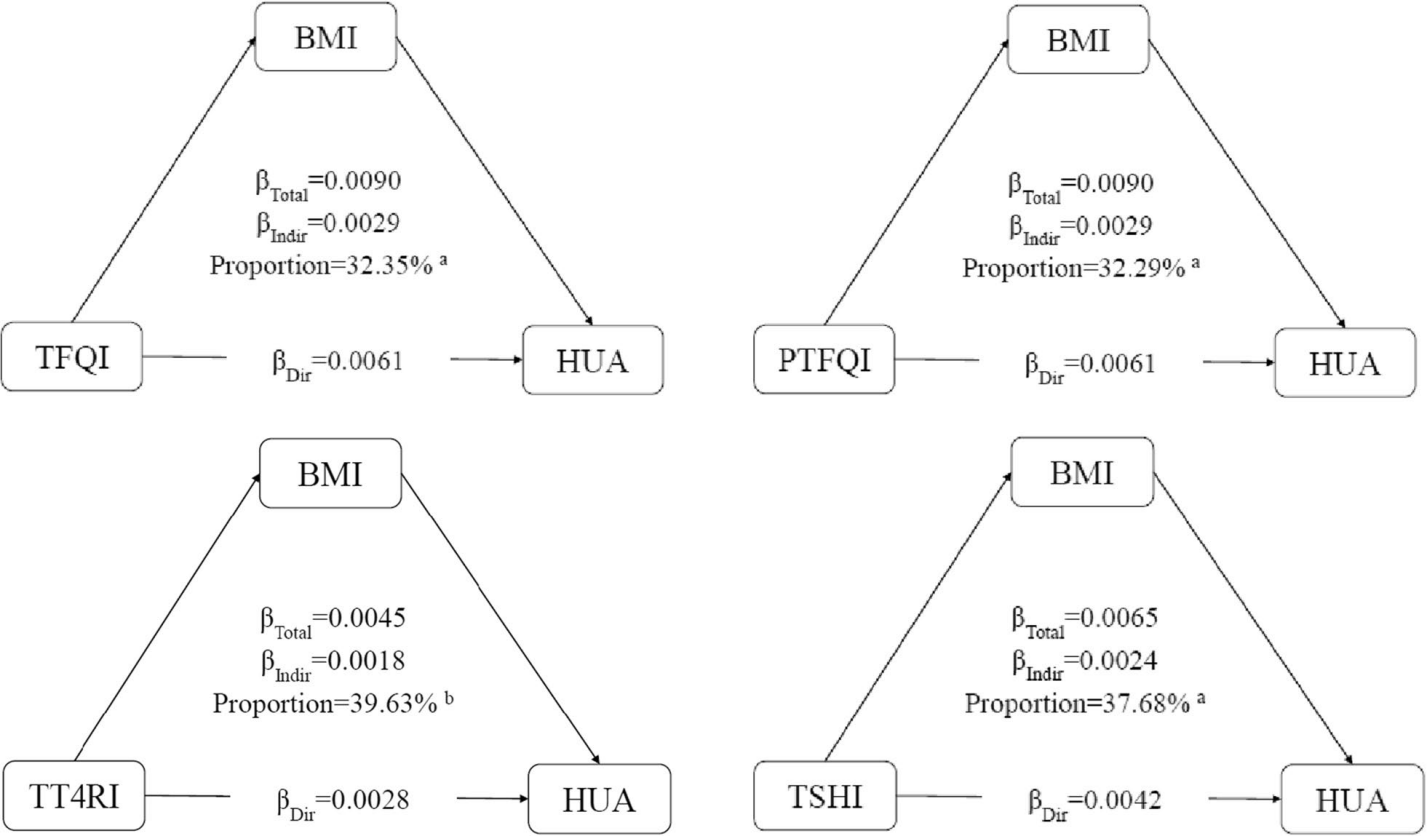
Mediation analyses of the association between continuous thyroid hormones sensitivity indices and hyperuricemia through BMI. *HUA* hyperuricemia, *BMI* body mass index, *TFQI* thyroid feedback quantile-based index, *PTFQI* parametric thyroid feedback quantile-based index, *TT4RI* thyrotrophic thyroxine resistance index, *TSHI* thyroid-stimulating hormone index. ^a^P value < 0.001, ^b^P value < 0.05

As shown in Additional file 1: Figure S4, there is a dose–response relationship between impaired sensitivity to thyroid hormones and obesity. Figure 2 summarizes the potential mediating effect of BMI between impaired sensitivity to thyroid hormones and hyperuricemia. The mediation proportion of TFQI was 32.35%, PTFQI was 32.29%, TT4RI was 39.63%, and TSHI was 37.68%. Additional file 1: Table S3 shows the direct and indirect coefficients between four indices of sensitivity to thyroid hormones and hyperuricemia through BMI. Consistent results were observed when the thyroid hormone sensitivity indices were considered categorical variables (Additional file 1: Table S4).

## Discussion

Using mediation analysis, we quantified the direct and indirect associations between impaired sensitivity to thyroid hormones and hyperuricemia through BMI in a large sample of euthyroid individuals. We found that impaired thyroid hormones sensitivity determined by the TFQI, PTFQI, TT4RI and TSHI was independently associated with prevalent hyperuricemia and that BMI significantly mediated the associations. To our knowledge, this is the first study to quantify the extent to which BMI mediates the association of impaired sensitivity to thyroid hormones with metabolic status. Our findings indicated that active weight control may be a practical intervention for individuals with impaired sensitivity to thyroid hormones to achieve metabolic health and homeostasis.

Physiologically, TSH and FT4 are regulated by a negative feedback mechanism in the hypothalamic-pituitary-thyroid axis. The cooccurrence of high TSH and high FT4 levels represents acquired resistance to thyroid hormones, which is a newly proposed clinical entity. In 2019, Laclaustra et al. [22] proposed the thyroid hormones sensitivity index (TFQI) to track the risk of metabolic syndrome, diabetes and diabetes-related mortality in euthyroid individuals. Previous studies have also confirmed the adverse effect of impaired sensitivity to thyroid hormones. For example, thyroid hormones resistance, as represented by TFQI, was significantly associated with an increased risk of diabetes and hypertension in euthyroid individuals according to a cross-sectional study [26]. In addition, a study showed that impaired sensitivity to thyroid hormones was associated with decreased kidney function [44]. A cross-sectional study of 8,957 adults with normal thyroid function reported that TFQI, PTFQI, TSHI and TT4RI were significantly associated with higher homocysteine levels [30]. A recent study reported a positive relationship between impaired sensitivity to thyroid hormones and hyperuricemia among individuals with subclinical hypothyroidism. Our findings supplemented the evidence that impaired sensitivity to thyroid hormones was also associated with hyperuricemia in euthyroid individuals, reinforcing an adverse effect of impaired sensitivity to thyroid hormones in the general population.

Previous studies on the separate relationships of TSH and FT4 with uric acid have yielded inconsistent results. One study found a negative correlation between TSH and uric acid [45], while another reported a positive association between elevated TSH levels and hyperuricemia regardless of age or sex [46]. On the other hand, a cross-sectional study [47] showed a linear positive association between FT4 level and serum uric acid among individuals without thyroid dysfunction. Our study highlighted the clinical importance of considering the interaction between TSH and FT4 (i.e., impaired sensitivity to thyroid hormones) on hyperuricemia and metabolic status, which partially explains the inconsistent findings from previous studies.

A recent study found a relationship between impaired sensitivity to thyroid hormones and obesity [27]. We investigated the direct and indirect effects of impaired thyroid hormones sensitivity on hyperuricemia through BMI. Our study indicated that BMI significantly mediated 32.3–39.6% of the associations between impaired sensitivity to thyroid hormones and hyperuricemia. Currently, there is no clinical consensus or intervention recommendations for individuals with impaired sensitivity to thyroid hormones. Our findings provide a practicable behavioral intervention through weight management for individuals with impaired sensitivity to thyroid hormones to achieve metabolic health. Several potential mechanisms may explain the association between thyroid hormones sensitivity and hyperuricemia mediated through BMI. Studies have demonstrated that serum TSH can promote weight gain, probably by stimulating preadipocyte differentiation and inducing adipogenesis [48, 49]. In addition, Nannipieri et al. [50] found that thyroid gene expression (especially TSH receptor) was reduced and plasma TSH concentration was higher among those with obesity. TSH exerts a beneficial effect on adipocytes through the TSH receptor to induce weight loss. These biological mechanisms indicate that weight intervention could potentially improve sensitivity to thyroid hormones. Moreover, aging could be another explanation linking thyroid hormones sensitivity and metabolic status [51, 52]. In recent years, the technique of mitochondria-targeting nanotechnology has provided broad prospects for the treatment of cancer and other mitochondria-related diseases (such as cardiovascular and neurological diseases), while the potential for impaired thyroid hormones sensitivity and hyperuricemia has not been studied. It is hoped that these advanced technologies could provide alternative therapies for metabolic health and hemostasis [53].

A major strength of this study relies on the large sample size. Individuals with higher levels of thyroid hormone sensitivity indices should be aware of the increased risk of hyperuricemia even with normal thyroid function. In addition, we estimated a potential pathway from impaired thyroid hormones sensitivity to hyperuricemia through BMI. To our knowledge, this is the first study to investigate the indirect effect of impaired thyroid hormones sensitivity on metabolic health through modifiable risk factors, which provided a behavioral recommendation through active weight management for individuals with impaired sensitivity to thyroid hormones. However, several limitations should be acknowledged. First, this cross-sectional study cannot establish longitudinal or causal associations of thyroid hormones sensitivity, BMI and hyperuricemia, and the possibility of reverse causality cannot be ruled out. Although we adjusted for several confounding factors, there could be residual confounding bias (e.g., thyroid-related antibody level) and recall bias in the effect estimation. Further studies (e.g., Mendelian randomization research) on the causal inference between thyroid hormones sensitivity and hyperuricemia are expected. Second, our study was based on the Chinese population, and the generalization of our findings requires further validation. The benefit of active weight management for individuals with impaired sensitivity to thyroid hormones should be evaluated in further clinical studies.

## Conclusions

Our study demonstrated that impaired sensitivity to thyroid hormones is associated with hyperuricemia in the euthyroid population and highlighted the mediating effect of BMI. These findings provide evidence for understanding the interaction between impaired sensitivity to thyroid hormones and hyperuricemia and suggest that active weight control among individuals with impaired thyroid hormones sensitivity may prevent or alleviate hyperuricemia, which warrants further clinical research.

## Supplementary Information

**Additional file 1: Table S1. **Basic characteristics of the participants according to obesity or not. **Table S2. **Logistic regression analysis for the association of thyroid hormone sensitivity and hyperuricemia among 28153 participants with available BMI data. **Table S3. **Mediation analyses of the association of continuous thyroid hormones sensitivity indices and hyperuricemia. **Table S4. **Estimated direct and indirect effect of thyroid hormones sensitivity indices (categorical) on hyperuricemia. **Figure S1. **Flow chart of this current study. **Figure S2. **The violin plot and boxplot in distribution of thyroid hormone sensitivity indices between two groups. **Figure S3.** Unadjusted and adjusted dose-response relationship between thyroid hormone sensitivity indices and hyperuricemia using restricted cubic spline method among 28153 participants with available BMI data. **Figure S4. **Dose-response relationship between thyroid hormone sensitivity indices and obesity using restricted cubic spline method among 28153 participants with available BMI data

## Data Availability

The datasets used and/or analysed during the current study are available from the corresponding authors on reasonable request.

## Abbreviations

BHMC: Beijing health management cohort
TSH: Thyrotropin/thyroid stimulating hormones
FT4: Free thyroxine
TFQI: Thyroid feedback quantile-based index
PTFQI: Parametric thyroid feedback quantile-based index
TT4RI: Thyrotrophic thyroxine resistance index
TSHI: Thyroid-stimulating hormone index
BMI: Body mass index
OR: Odds ratio
ARD: Absolute risk difference
CI: Confidence interval
