# Supplementary material for: Association of impaired sensitivity to thyroid hormones with hyperuricemia through obesity in the euthyroid population
Source: J Transl Med. 2023 Jul 5;21:436. doi: 10.1186/s12967-023-04276-3 (PMC10320931; doi:10.1186/s12967-023-04276-3)
Supplement: Supplementary file 1 — Additional file 1: Table S1. Basic characteristics of the participants according to obesity or not. Table S2. Logistic regression analysis for the association of thyroid hormone sensitivity and hyperuricemia among 28153 participants with available BMI data. Table S3. Mediation analyses of the association of continuous thyroid hormones sensitivity indices and hyperuricemia. Table S4. Estimated direct and indirect effect of thyroid hormones sensitivity indices (categorical) on hyperuricemia. Figure S1. Flow chart of this current study. Figure S2. The violin plot and boxplot in distribution of thyroid hormone sensitivity indices between two groups. Figure S3. Unadjusted and adjusted dose-response relationship between thyroid hormone sensitivity indices and hyperuricemia using restricted cubic spline method among 28153 participants with available BMI data. Figure S4. Dose-response relationship between thyroid hormone sensitivity indices and obesity using restricted cubic spline method among 28153 participants with available BMI data [file 12967_2023_4276_MOESM1_ESM.docx]

**Additional Material**

**Association of impaired sensitivity to thyroid hormones with hyperuricemia through obesity in the euthyroid population**

Zhiyuan Wu, et al.

**1 Additional Tables**

**Table S1:** Basic characteristics of the participants according to obesity or not

**Table S2:** Logistic regression analysis for the association of thyroid hormone sensitivity and hyperuricemia among 28153 participants with available BMI data

**Table S3:** Mediation analyses of the association of continuous thyroid hormones sensitivity indices and hyperuricemia

**Table S4:** Estimated direct and indirect effect of thyroid hormones sensitivity indices (categorical) on hyperuricemia

**2 Additional Figures**

**Figure S1:** Flow chart of this current study

**Figure S2:** The violin plot and boxplot in distribution of thyroid hormone sensitivity indices between two groups

**Figure S3:** Unadjusted and adjusted dose-response relationship between thyroid hormone sensitivity indices and hyperuricemia using restricted cubic spline method among 28153 participants with available BMI data

**Figure S4:** Dose-response relationship between thyroid hormone sensitivity indices and obesity using restricted cubic spline method among 28153 participants with available BMI data

**Table S1** Basic characteristics of the participants according to obesity or not

|  | **Overall** | **Non-obesity** | **Obesity** ^f^ | ***P* value** |
| --- | --- | --- | --- | --- |
| Participants, No. | 28153 | 22497 | 5656 |  |
| Age, mean (SD), y | 47.4 (13.4) | 47.3 (13.5) | 48.0 (13.0) | **<0.001** |
| Sex |  |  |  | **<0.001** |
| Female | 10548 (37.5) | 9424 (41.9) | 1124 (19.9) |  |
| Male | 17065 (62.5) | 13073 (58.1) | 4532 (80.1) |  |
| Educational level |  |  |  | **<0.001** |
| Primary | 2570 (9.1) | 1984 (8.8) | 586 (10.4) |  |
| Secondary | 19956 (70.9) | 15942 (70.9) | 4014 (71.0) |  |
| Tertiary | 5627 (20.0) | 4571 (20.3) | 1056 (18.7) |  |
| Active physical activity ^a^ | 11373 (40.4) | 9157 (40.7) | 2216 (39.2) | **0.038** |
| Current smoking | 7278 (25.9) | 5517 (24.5) | 1761 (31.1) | **<0.001** |
| Current drinking | 14610 (51.9) | 11418 (50.8) | 3192 (56.4) | **<0.001** |
| BMI, mean (SD), kg/m^2 b^ | 25.3 (3.5) | 24.0 (2.5) | 30.4 (2.3) | **<0.001** |
| Hypertension ^c^  Diabetes ^d^ | 6071 (21.6)  2864 (10.2) | 3946 (17.5)  1935 (8.6) | 2125 (37.6)  929 (16.4) | **<0.001**  **<0.001** |
| TSH, median [IQR], mIU/L | 1.9 [1.4, 2.7] | 1.9 [1.4, 2.7] | 2.0 [1.4, 2.7] | **0.004** |
| FT4, median [IQR], pmol/L | 15.8 [14.3, 17.4] | 15.8 [14.2, 17.4] | 15.9 [14.3, 17.4] | **0.040** |
| UA, mean (SD), μmol/L | 341.0 (89.4) | 329.4 (85.6) | 386.9 (89.3) | **<0.001** |
| Hyperuricemia ^e^ | 5968 (21.2) | 3889 (17.3) | 2079 (36.8) | **<0.001** |

Abbreviations: SD, standard deviation; IQR, interquartile range; BMI, body mass index; TSH, thyrotropin; FT4, free thyroxine; UA, uric acid.

SI conversion factors: To convert FT4 to ng/dL, divided by 12.871.

^a^ Active physical activity refers to having moderate or intense exercise ≥80 minutes a weak.

^b^ BMI is calculated as weight in kilograms divided by height in meters squared. Data of BMI were available among 28153 participants.

^c^ Hypertension was defined as systolic blood pressure ≥140 mmHg or diastolic blood pressure ≥90 mmHg or self-reported diagnosis history of hypertension or use of any anti-hypertensive medication.

^d^ Diabetes was defined as fasting glucose ≥7.0 mmol/L or self-reported diagnosis history of diabetes or using any glucose-lowering medication.

^e^ Hyperuricemia was defined as serum uric acid ≥360 μmol/L in female and ≥420 μmol/L in male or using uric acid lowering medications.

f Obesity was defined using BMI ≥ 28.0 kg/m^2^ for the Chinese population.

**Table S2** Logistic regression analysis for the association of thyroid hormone sensitivity and hyperuricemia among 28153 participants with available BMI data

|  | **Model 1** | |  | **Model 2** | |
| --- | --- | --- | --- | --- | --- |
|  | **OR (95% CI)** | **P value** |  | **OR (95% CI)** | ***P* value** |
| TFQI |  |  |  |  |  |
| Group 1 | 1 [Ref] |  |  | 1 [Ref] |  |
| Group 2 | 1.048 (0.981-1.120) | 0.166 |  | 1.020 (0.952-1.092) | 0.579 |
| Group 3 | 1.119 (1.033-1.212) | **0.006** |  | 1.023 (0.942-1.111) | 0.592 |
| Group 4 | 1.354 (1.184-1.549) | **<0.001** |  | 1.141 (0.994-1.127) | 0.061 |
| PTFQI |  |  |  |  |  |
| Group 1 | 1 [Ref] |  |  | 1 [Ref] |  |
| Group 2 | 1.052 (0.984-1.124) | 0.138 |  | 1.024 (0.956-1.097) | 0.495 |
| Group 3 | 1.114 (1.029-1.205) | **0.008** |  | 1.017 (0.938-1.104) | 0.680 |
| Group 4 | 1.379 (1.209-1.573) | **<0.001** |  | 1.151 (1.005-1.318) | **0.042** |
| TSHI |  |  |  |  |  |
| Quartile 1 | 1 [Ref] |  |  | 1 [Ref] |  |
| Quartile 2 | 0.996 (0.917-1.081) | 0.916 |  | 1.018 (0.936-1.108) | 0.677 |
| Quartile 3 | 1.056 (0.973-1.145) | 0.191 |  | 1.075 (0.989-1.169) | 0.088 |
| Quartile 4 | 1.136 (1.048-1.231) | **0.002** |  | 1.108 (1.020-1.204) | **0.015** |
| TT4RI |  |  |  |  |  |
| Quartile 1 | 1 [Ref] |  |  | 1 [Ref] |  |
| Quartile 2 | 0.993 (0.916-1.078) | 0.876 |  | 1.015 (0.934-1.104) | 0.721 |
| Quartile 3 | 1.042 (0.961-1.131) | 0.315 |  | 1.094 (1.006-1.189) | **0.036** |
| Quartile 4 | 1.086 (1.002-1.177) | **0.044** |  | 1.164 (1.071-1.265) | **<0.001** |

Abbreviations: OR, odds ratio; CI, confidence interval; TFQI, thyroid feedback quantile-based index; PTFQI, parametric thyroid feedback quantile-based index; TT4RI, thyrotrophic thyroxine resistance index; TSHI, thyroid-stimulating hormone index.

Model 1: crude model; model 2: adjusted for sex, age (continuous), education level (categorical), current smoking (categorical), current drinking (categorical), active physical activity (categorical), hypertension (categorical) and diabetes (categorical).

**Table S3** Mediation analyses of the association of continuous thyroid hormones sensitivity indices and hyperuricemia

|  | **β (95% CI)** | | | **Mediated**  **Proportion** |
| --- | --- | --- | --- | --- |
|  | **Total Effect** | **Direct Effect** | **Indirect Effect** |  |
| TFQI | 0.0090 (0.0043 to 0.0138) | 0.0061 (0.0020 to 0.0112) | 0.0029 (0.0017 to 0.0040) | **32.35% ^a^** |
| PTFQI | 0.0090 (0.0043 to 0.0139) | 0.0061 (0.0020 to 0.0112) | 0.0029 (0.0017 to 0.0040) | **32.29% ^a^** |
| TT4RI | 0.0045 (-0.0001 to 0.0093) | 0.0028 (-0.0012 to 0.0078) | 0.0018 (0.0006 to 0.0029) | **39.63% ^b^** |
| TSHI | 0.0065 (0.0018 to 0.0113) | 0.0042 (0.0001 to 0.0093) | 0.0024 (0.0012 to 0.0035) | **37.68% ^a^** |

Abbreviations: CI, confidence interval; TFQI, thyroid feedback quantile-based index; PTFQI, parametric thyroid feedback quantile-based index; TT4RI, thyrotrophic thyroxine resistance index; TSHI, thyroid-stimulating hormone index.

^a^ P value <0.001.

^b^ P value <0.05.

**Table S4** Estimated direct and indirect effect of thyroid hormones sensitivity indices (categorical) on hyperuricemia

|  | **HUA, No./**  **Total No.** | **β (95% CI) ^a^** | | | **% Mediated** |
| --- | --- | --- | --- | --- | --- |
|  |  | **Total Effect** | **Direct Effect** | **Indirect Effect** |  |
| TFQI |  |  |  |  |  |
| Group 1 | 2738 / 13420 | [Ref] | [Ref] | [Ref] |  |
| Group 2 | 1843 / 8704 | 0.0074 (-0.0035 to 0.0201) | 0.0035 (-0.0070 to 0.0157) | 0.0039 (0.0014 to 0.0060) | 53.10 |
| Group 3 | 1069 / 4795 | 0.0187 (0.0040 to 0.0332) | 0.0126 (-0.0015 to 0.0225) | 0.0061 (0.0020 to 0.0093) | 32.54 ^b^ |
| Group 4 | 318 / 1234 | 0.0520 (0.0299 to 0.0785) | 0.0430 (0.0228 to 0.0692) | 0.0090 (0.0025 to 0.0161) | 17.31 ^b^ |
| PTFQI |  |  |  |  |  |
| Group 1 | 2711 / 13310 | [Ref] | [Ref] | [Ref] |  |
| Group 2 | 1825 / 8609 | 0.0080 (-0.0018 to 0.0214) | 0.0039 (-0.0057 to 0.0170) | 0.0041 (0.0017 to 0.0065) | 51.06 |
| Group 3 | 1098 / 4953 | 0.0177 (0.0039 to 0.0313) | 0.0117 (-0.0013 to 0.0245) | 0.0059 (0.0022 to 0.0092) | 33.62 ^b^ |
| Group 4 | 334 / 1281 | 0.0558 (0.0329 to 0.0822) | 0.0457 (0.0224 to 0.0719) | 0.0101 (0.0035 to 0.0170) | 18.06 ^b^ |
| TT4RI |  |  |  |  |  |
| Quartile 1 | 1453 / 7018 | [Ref] | [Ref] | [Ref] |  |
| Quartile 2 | 1449 / 7035 | -0.0010 (-0.0127 to 0.0141) | -0.0008 (-0.0120 to 0.0120) | -0.0002 (-0.0036 to 0.0034) | 20.49 |
| Quartile 3 | 1509 / 7053 | 0.0060 (-0.0056 to 0.0185) | 0.0032 (-0.0077 to 0.0152) | 0.0027 (-0.0006 to 0.0066) | 45.96 |
| Quartile 4 | 1557 / 7047 | 0.0128 (0.0023 to 0.0255) | 0.0075 (-0.0031 to 0.0201) | 0.0053 (0.0021 to 0.0091) | 41.29 ^b^ |
| TSHI |  |  |  |  |  |
| Quartile 1 | 1435 / 7016 | [Ref] | [Ref] | [Ref] |  |
| Quartile 2 | 1431 / 7021 | -0.0010 (-0.0116 to 0.0144) | -0.0023 (-0.0117 to 0.0115) | 0.0013 (-0.0018 to 0.0047) | -129.42 |
| Quartile 3 | 1509 / 7068 | 0.0083 (-0.0049 to 0.0229) | 0.0033 (-0.0088 to 0.0182) | 0.0049 (0.0012 to 0.0087) | 59.51 |
| Quartile 4 | 1593 / 7048 | 0.0207 (0.0093 to 0.0330) | 0.0142 (0.0027 to 0.0263) | 0.0065 (0.0031 to 0.0100) | 31.30 ^b^ |

Abbreviations: HUA, hyperuricemia; CI, confidence interval; TFQI, thyroid feedback quantile-based index; PTFQI, parametric thyroid feedback quantile-based index; TT4RI, thyrotrophic thyroxine resistance index; TSHI, thyroid-stimulating hormone index.

^a^ Refers to the pairwise comparison with group1 or quartile 1 as the reference group.

^b^ P value < 0.05.

**Supplemental Figures**


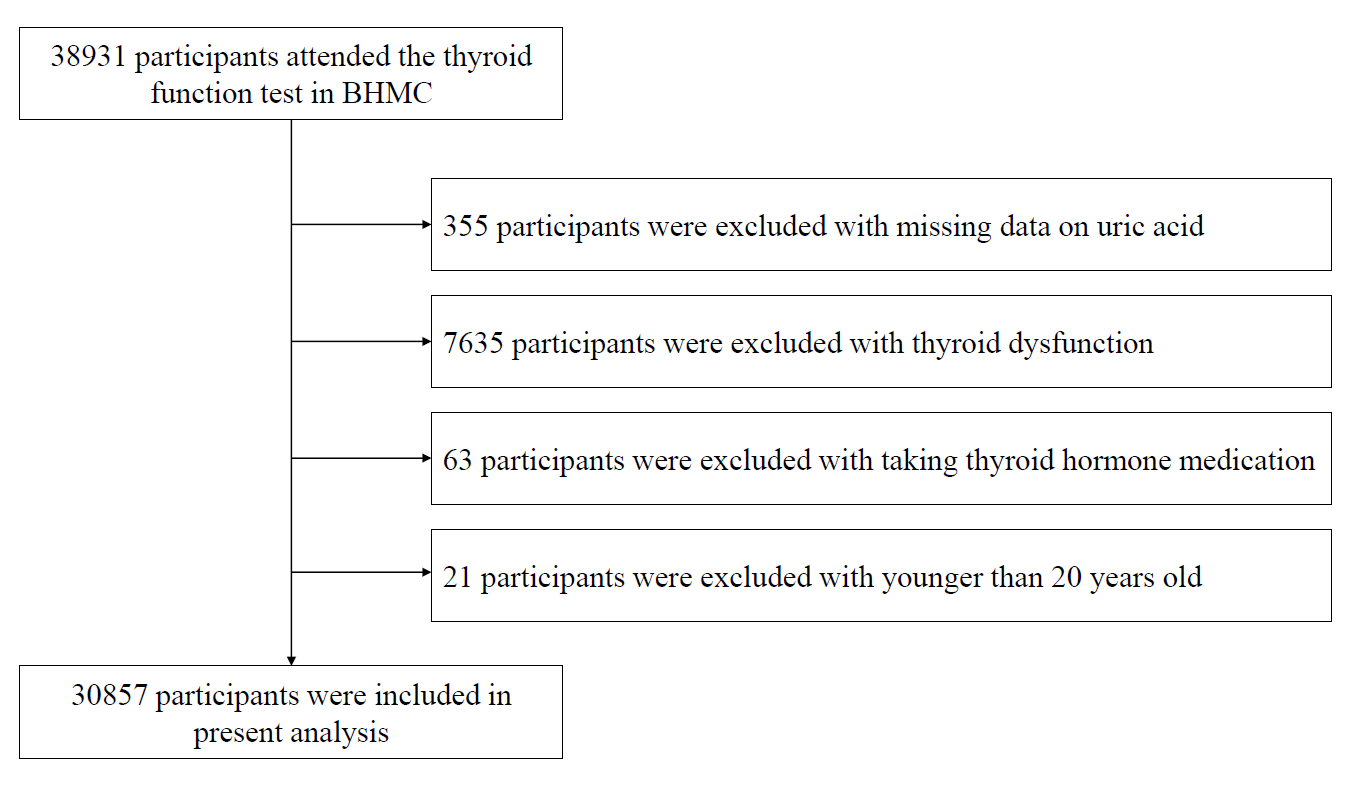


**Figure S1** Flow chart of this current study

Abbreviations: BHMC, Beijing health management cohort.


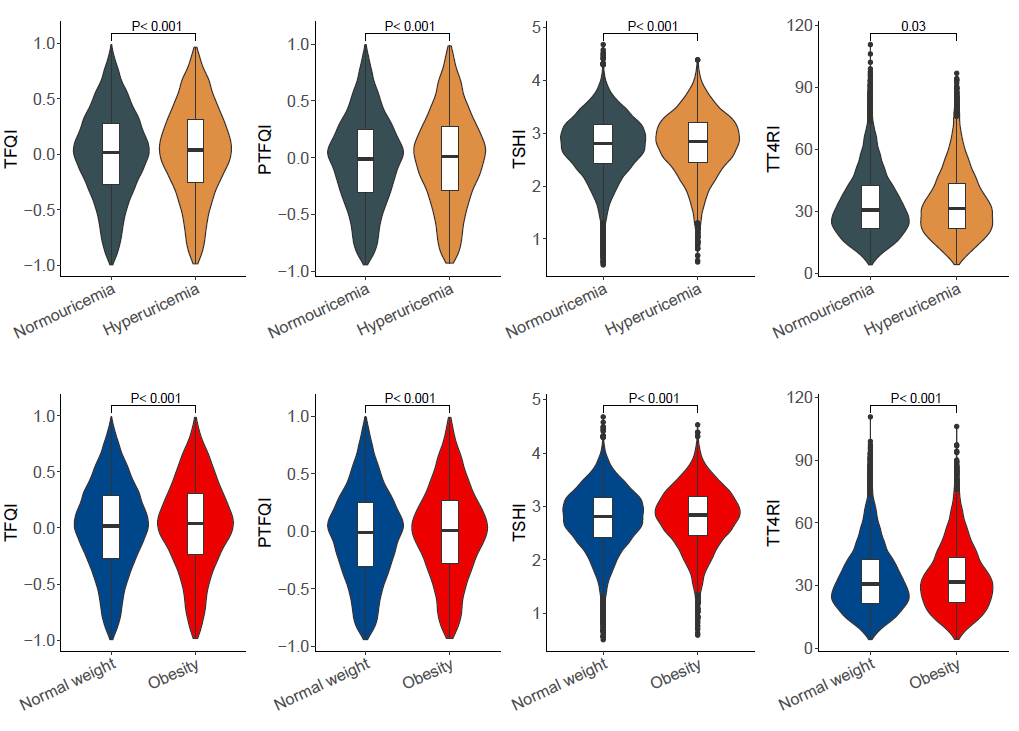


**Figure S2** The violin plot and boxplot in distribution of thyroid hormone sensitivity indices between two groups

Abbreviations: TFQI, thyroid feedback quantile-based index; PTFQI, parametric thyroid feedback quantile-based index; TT4RI, thyrotrophic thyroxine resistance index; TSHI, thyroid-stimulating hormone index.

Obesity was defined using BMI ≥ 28.0 kg/m^2^.


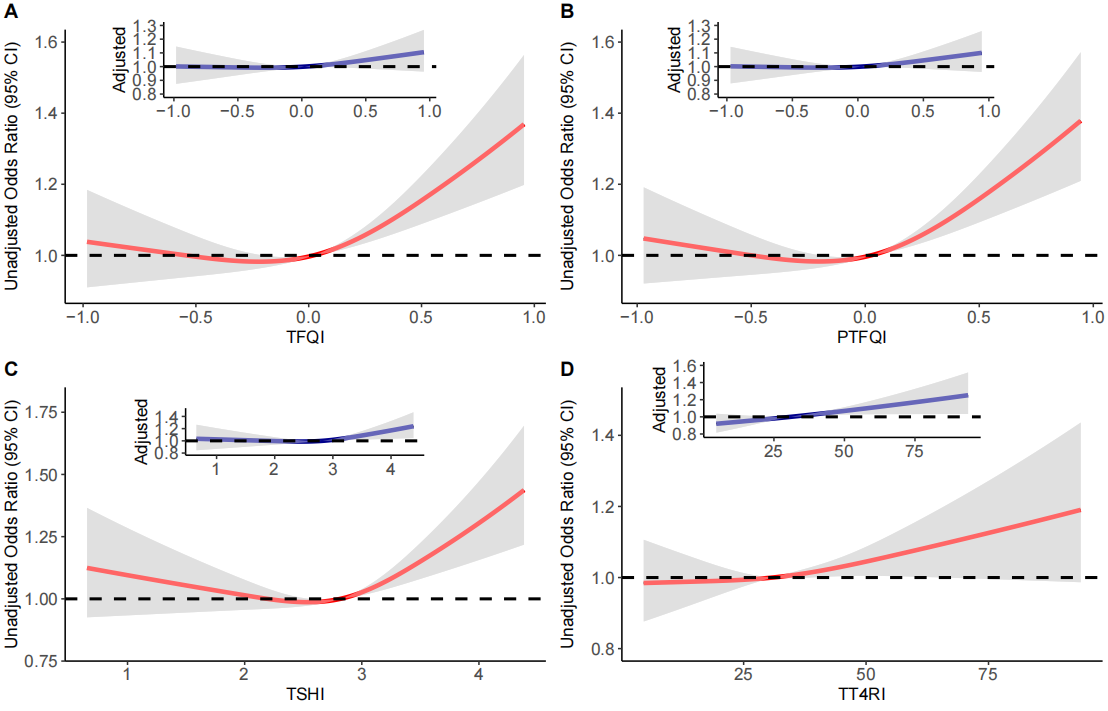


**Figure S3** Unadjusted and adjusted dose-response relationship between thyroid hormone sensitivity indices and hyperuricemia using restricted cubic spline method among 28153 participants with available BMI data

Restricted cubic spline regression model was conducted using 3 knots at the 10th, 50th, and 90th percentiles; results were adjusted for sex, age (continuous), education level (categorical), current smoking (categorical), current drinking (categorical), active physical activity (categorical), hypertension (categorical) and diabetes (categorical).

Abbreviations: BMI, body mass index; TFQI, thyroid feedback quantile-based index; PTFQI, parametric thyroid feedback quantile-based index; TT4RI, thyrotrophic thyroxine resistance index; TSHI, thyroid-stimulating hormone index.


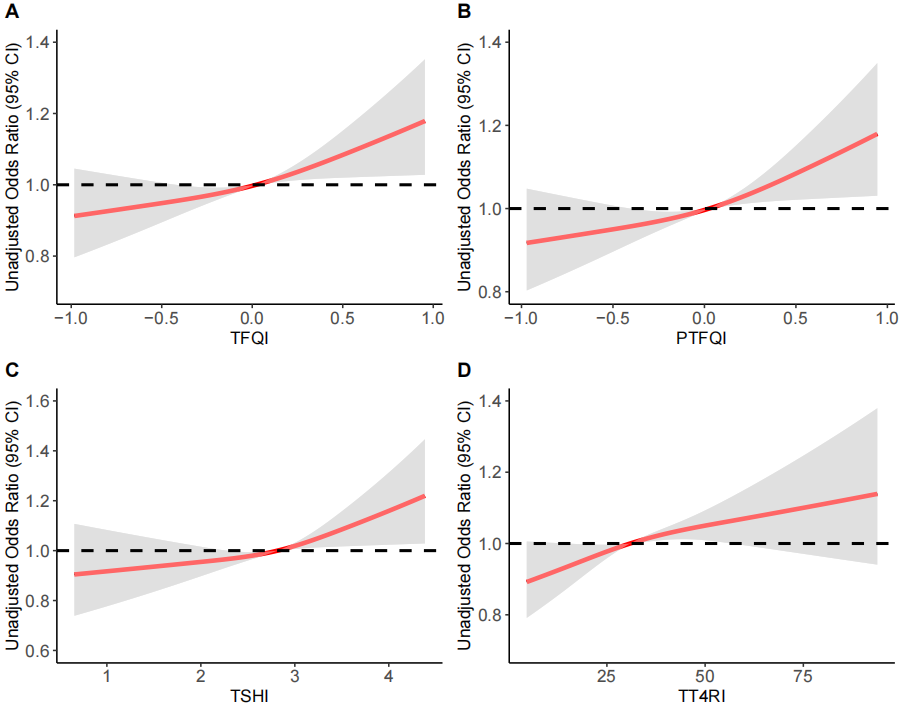


**Figure S4** Dose-response relationship between thyroid hormone sensitivity indices and obesity using restricted cubic spline method among 28153 participants with available BMI data

Abbreviations: BMI, body mass index; TFQI, thyroid feedback quantile-based index; PTFQI, parametric thyroid feedback quantile-based index; TT4RI, thyrotrophic thyroxine resistance index; TSHI, thyroid-stimulating hormone index.

Obesity was defined using BMI ≥ 28.0 kg/m^2^.
